# Risk and outcome of pyelonephritis among renal transplant recipients

**DOI:** 10.1186/s12879-016-1608-x

**Published:** 2016-06-10

**Authors:** Mette Elneff Graversen, Lars Skov Dalgaard, Søren Jensen-Fangel, Bente Jespersen, Lars Østergaard, Ole Schmeltz Søgaard

**Affiliations:** Department of Infectious Diseases, Aarhus University Hospital, Palle Juul-Jensens Boulevard 99, 8200 Aarhus N, Denmark; Department of Renal Medicine, Aarhus University Hospital, Aarhus N, Denmark

**Keywords:** End-stage renal disease, Renal transplantation, Pyelonephritis, Graft loss, Mortality, Hospitalisation

## Abstract

**Background:**

Urinary tract infection is the most common infectious disease requiring hospitalisation following renal transplantation. However, the risk and outcome of post-transplant pyelonephritis remains unclear.

**Methods:**

This population-based cohort study was conducted from 1 January 1990 to 31 December 2009. Each member of a Danish population-based, nationwide cohort of first-time renal transplant recipients was matched by age and gender with up to 19 population controls. Information on hospital discharge diagnosis, emigration, and mortality was obtained from nationwide administrative databases. Individuals were observed from the date of first renal transplantation and until graft loss, emigration, or death. Risk factors were assessed by Poisson regression.

**Results:**

The incidence rate (IR) of first-time hospitalisation for pyelonephritis was 18.5 (95 % confidence interval [CI]: 16.4–20.9) per 1,000 person-years of follow-up (PYFU) among renal transplant recipients (*N* = 2,656) and 0.26 (CI: 0.21–0.31) per 1,000 PYFU among population controls (*N* = 49,226) yielding an incidence rate-ratio (IRR) of 72.0 (95 % CI: 57.8–89.7). Among renal transplant recipients, the risk of pyelonephritis decreased during the entire study period and was lowest in 2005–09 (IRR = 0.46, CI: 0.31–0.68). The highest risk of pyelonephritis was observed within the first six months post-transplantation (IR = 69.9 per 1,000 PYFU; CI: 56.4–86.7). Other risk factors for post-transplant pyelonephritis included female gender, high Charlson comorbidity index score, HLA-DR mismatch, cause of renal failure, and calendar period. Interestingly, we found that the combined risk of graft loss and death was 45 %, (CI: 19–77 %) higher in renal transplant recipients following post-transplant pyelonephritis compared to those who had no admission due to pyelonephritis.

**Conclusions:**

The risk of first-time hospitalisation for pyelonephritis among renal transplant recipients is high. Further, post-transplant pyelonephritis was associated with excess risk of graft loss and death; this indicates that strategies aimed at reducing upper urinary tract infections are likely to enhance renal graft survival.

## Background

Renal transplantation dramatically improves quality of life and life expectancy compared to conventional hemodialysis in end-stage renal disease (ESRD). In 2010, annual renal transplant rates in the United Stated and Denmark were 57.5 and 41.3 per million population, respectively [1].

Immunosuppressive therapy in conjunction with per- and post-operative instrumentation of the urinary tract, renders renal transplant (RTx) recipients prone to infectious complications [2]. In addition, RTx recipients often have various co-morbidities such as cardiovascular disease and diabetes mellitus, further increasing their risk of infection.

The most common bacterial infection following renal transplantation is urinary tract infection (UTI) including pyelonephritis [3, 4], ranging from 7 to 86 % [5, 6]. The incidence of UTI among RTx recipients has declined during the past three decades [7]. However, previous studies have shown that RTx recipients may have an increased risk of UTI compared to the background population [8–10]. It remains controversial whether UTI causes RTx recipients to be prone to graft loss and/or associated with an increased mortality rate.

The purpose of this nationwide population-based study was to determine the incidence of pyelonephritis and to identify risk factors for post-transplant pyelonephritis among RTx recipients and population controls, as well as to determine impact on graft survival and mortality in RTx recipients.

## Methods

### Study design and setting

We conducted a nationwide population based cohort study among RTx recipients in Denmark between 1990 and 2009. In the study period, treatment of ESRD in Denmark was offered at 15 specialised nephrology departments of which four performed renal transplantations. Each department reported data to the Danish Nephrology Registry (DNR) database [11], which has collected nationwide data with a completeness of 97.2 % on patients with ESRD since 1990 [12]. In 2011, the DNR database included data on approximately 15,000 patients with ESRD of which 4,062 received a renal graft between 1990 and 2009.

### Study population

We identified RTx recipients at 16 years or older receiving their first transplant after 1 January 1990 in the DNR database. For each RTx recipient we sampled up to 19 controls matched on gender and age (month and year of birth) on the day the RTx patient was diagnosed with ESRD using the Danish Civil Registration System (CRS). Since 1968, all Danish citizens have been registered in the CRS [13] encompassing information on date of birth, age, and gender, through the unique 10-digit civil registration number (CRN) assigned to each citizen. The CRS was further used to obtain information on date of migration and death. The CRN enables accurate linkage across databases.

### Identification of hospitalisation of patients with pyelonephritis

The Danish National Registry of Patients (DNPR) has collected data on all patients admitted to Danish somatic hospitals since 1977. Since 1995, data on attendance to emergency and outpatient clinics has also been included. The DNPR contains information on e.g., the CRN, diagnosis codes and dates of admission and discharge. Since 1994 the diagnoses have been coded in accordance with the 10th edition of the International Classification of Diseases (ICD-10). From 1977 to 1993, the diagnoses were coded with reference to the 8th edition (ICD-8). Using data from DNPR, all hospital admissions with a discharge diagnosis of pyelonephritis with or without known renal disease were identified. In this study, ICD-8 (59010) and ICD-10 (DN109 and DN129) were used to identify hospitalisation of patients with pyelonephritis. Patients with a prior discharge diagnosis of pyelonephritis were excluded.

### Validation of the pyelonephritis diagnosis

To validate the diagnosis of pyelonephritis in the DNPR we reviewed a representative sample of 75 medical records from patients with pyelonephritis as the primary discharge diagnosis in the study period from 1990 to 2009. The reviews were confined to Aarhus University Hospital, since the data quality in the DNPR is considered to be uniform throughout the country [14].

### Definition of pyelonephritis

Pyelonephritis is a clinical syndrome which lacks a uniformly accepted definition.

In the validation process, we used the following criteria to confirm the pyelonephritis diagnosis in the DNRP (at least one positive finding from each of the three following categories was required):

1. Positive microbiology or imaging,
  - comprising a urinary stix positive for nitrite and/or leukocyte esterase and/or a positive urine culture and/or ultrasound marks indicating pyelonephritis.
2. Positive laboratory test results
  - including leukocyte count ≥ 12x 10^9^ cells/L and/or CRP ≥ 80 mg/L and/or temperature > 37.8°∁.
3. Positive clinical signs,
  - including one or more of the following: graft-pain/flank pain, chills, clinical symptoms consistent with cystitis (dysuria, pollakisuria, urge, suprapubic pain).

### Statistical analysis

#### Time at risk

For patients with ESRD, time at risk was calculated from the date of first renal transplantation and until graft loss, lost to emigration, death, or 31 December 2009 (end of study period), whichever came first. Population controls entered the study on the same day as their matched RTx recipient.

#### Incidence rate, incidence rate-ratio and cumulative incidence

Four calendar periods were defined (1990–94, 1995–99, 2000–04, and 2005–09) and incidence rates (IRs) of first-time hospital admission for pyelonephritis were calculated for RTx recipients and population controls for each of the calendar periods and compared by calculating the incidence rate-ratio (IRR) with 95 % confidence intervals (CI). IRRs were calculated for the whole study period with and without adjustment for comorbidity, calendar periods, age, and gender.

The cumulative incidence of first time hospitalisation for pyelonephritis was calculated for both RTx recipients and population controls stratified by gender.

#### Comorbidity data

The Charlson comorbidity index (CCI) score for each study participant based on the complete hospital discharge history was calculated on the date of their study entry. The CCI includes 19 major disease categories and has been adapted and validated for use with hospital discharge data in ICD databases. The CCI is considered a reliable method for measuring comorbidity in clinical research [15]. In this study, the CCI score did not include renal disease.

#### Risk factors

Poisson regression was used to assess risk factors for first time hospitalisation for pyelonephritis in RTx recipients. The variables included were gender, CCI score (low (0), medium (1–2), and high (≥3)), donor type (cadaveric donor, living unrelated donor, and living related donor), human leukocyte antigen (HLA) mismatches (HLA AB mismatches <3/≥3 and HLA DR mismatches <2/2), cause of ESRD (glomerulonephritis, diabetes mellitus types I and II, chronic interstitial nephritis, hypertensive kidney disease, polycystic kidney disease, and vasculitis), time since transplantation (0–6, 7–12, 13–24, and >24 months), recipient age (16–49 years, 50–64 years, and ≥65 years), and calendar period.

#### Mortality and risk of graft loss

We used 90-day mortality to compare survival among RTx recipients and population controls following diagnosis of pyelonephritis. The potential association between hospitalisation for pyelonephritis and the combined risk of graft loss and death in RTx recipients was investigated using poisson regression with pyelonephritis as exposure (time-varying covariate). To exclude potential bias from early graft loss, we conducted sensitivity analyses in which the first 90 days post-transplantation were censored. Both of these analyses were adjusted for potential confounders (sex and age).

Stata software, version 11.0 (StataCorp, College Station, Texas) was used for statistical analyses.

## Results

### Study population

The study population consisted of 2,656 RTx recipients and 49,226 matched population controls, yielding 14,121 and 447,976 person years of follow-up (PYFU), respectively. Median follow-up period was 4.3 years for RTx recipients and 8.7 years for population controls. A first-time hospitalisation for pyelonephritis was identified in 261 RTx recipients (9.8 %) and 115 population controls (0.2 %).

### Incidence of pyelonephritis

The overall IR of first-time hospitalisation for pyelonephritis in the entire study period was 18.5 (CI: 16.4–20.9) per 1,000 PYFU among RTx recipients, and 0.26 (CI: 0.21–0.31) per 1,000 PYFU among population controls. A decrease in IR of first-time hospitalisation for pyelonephritis was observed among RTx recipients from 1990 to 2009, with a maximum of 30.0 hospitalisations per 1,000 PYFU (CI: 21.0–42.0) in 1990–94, and a minimum of 14.0 hospitalisations per 1,000 PYFU (CI: 11.0–17.0) in 2005–09 (Table 1). The relative risk of hospitalisation for pyelonephritis among RTx recipients was 0.46 (CI: 0.31–0.68) in 2005–09 compared to 1990–94.

**Table 1:** Incidence of first hospitalization for pyelonephritis in renal transplant recipients

Characteristic	Pyelonephritis Hospitalizations, No.^a^	Person-Years Follow up	IR (95 % Cl)	IRR (95 % Cl)	*P* ^*^ Value
Entire study period (1990–09)					
Renal transplant recipients	261	14,121	18.5 (16.4–20.9)	772.0 (57.8–89.7)	<.001
Population controls	115	447,976	0.26 (0.21–0.31)		
1990–1994:					
Renal transplant recipients	35	1,175	30.0 (21.0–42.0)	841 (115–-6138)	<.001
Population controls	1	28,218	0.04 (0.01─0.25)		
1995–1999:					
Renal transplant recipients	67	2,974	23.0 (18.0–29.0)	178 (94.1–337)	<.0001
Population controls	11	86,904	0.13 (0.07–0.23)		
2000–2004:					
Renal transplant recipients	81	4,242	19.0 (15.0–24.0	75.6 (50.8–112)	<.0001
Population controls	35	138,543	0.25 (0.18–0.35)		
2005–2009:					
Renal transplant recipients	78	5,731	14.0 (11.0–17.0)	38.9 (28.1–53.8)	<.0001
Population controls	68	194,311	0.35 (0.28–0.44)		
Male:					
Renal transplant recipients	122	9,373	13.0 (10.9–15.5)	69.4(50.3–95.8)	<.0001
Population controls	53	282,662.3	0.19 (0.14–0.25)		
Female:					
Renal transplant recipients	139	4,747.2	29.3(24.8–34.6)	78.1(57.9–105)	<.0001
Population controls	62	165,314.9	0.38(0.29─0.48)		
<50 years					
Renal transplant recipients	140	7,696	18.2(15.4–21.5)	117 (79.8–170)	<.0001
Population controls	33	211,410	0.16(0.11–0.22)		
50–65 years:					
Renal transplant recipients	98	5,235	18.7(15.4–22.8)	81.7 (56.8–118)	<.0001
Population controls	41	178,960	0.23(0.17–0.31)		
65 + years:					
Renal transplant recipients	23	1,190	19.3(12.8–29.1)	127.2 (16.3–45.2)	<.0001
Population controls	41	57,607	0.71(0.52–0.97)		

*Abbreviations*: *Cl* confidence interval, *IR* incidence rate, *IRR* incidence rate ratio

^*^ *P* value corresponding to IRR

^a^No. of hospitalizations per 1000 person-years

The unadjusted risk of pyelonephritis among RTx recipients was very high compared to population controls (IRR = 72.0, CI: 57.8–89.7). Adjusting for calendar periods, age, gender and CCI score, only resulted in a modest change in the relative risk of pyelonephritis among RTx recipients (adjusted IRR = 63.9, CI: 50.2–81.3). The majority of hospitalisations for pyelonephritis (31.8 %) among RTx recipients were seen within the first six months post-transplantation.

### Impact of comorbidity on risk of pyelonephritis

The risk of first-time hospitalisation for pyelonephritis was considerably higher in RTx recipients with a medium or high CCI score than in those with low CCI score (Table 2). Comparing RTx recipients without comorbidity (other than ESRD) and population controls without comorbidity yielded an IRR of 73.5 (CI: 56.1–96.3).

**Table 2:** Incidence rates and rate-ratios for pyelonephritis according to the burden of comorbidity

Subgroup	CCI-score^a^	No.^b^ (%)	PN, hospitalizations, No.^c^	IR(95 % CI)	IRR (95 % CI)	*P* value
Renal transplant recipients	Low (0)	1,393 (52.5)	131	15.9 (13.4–18.9)	73.5 (56.1–96.3)	<.0001
Population controls	Low (0)	43,253 (87.9)	88	0.22 (0.18–0.27)	1 (Reference)	
Renal transplant recipients	Medium (1–2)	707 (26.6)	60	17.8 (13.9–23.0)	36.9 (21.5–63.2)	<.0001
Population controls	Medium (1–2)	4,954 (10.1)	17	0.48 (0.30–0.78)	1 (Reference)	
Renal transplant recipients	High ≥ 3	556 (20.9)	70	27.9 (22.0–35.2)	16.0 (8.2–31.0)	<.0001
Population controls	High ≥ 3	1,019 (2.1)	10	1.74 (0.94–3.24)	1 (Reference)	

*Abbreviations*: *CI* confidence interval, *IR* incidence rate, *IRR* incidence rate ratio

^a^Level of Charlson co-morbidity index score (see text)

^b^Number and percentage allocated on the CCI-score

^c^No. of Hospitalizations per 1000 person-years

### Risk factors for hospitalisation for pyelonephritis among RTx recipients

In RTx recipients risk factors for first-time hospitalisation for pyelonephritis in the adjusted analysis were female gender (IRR = 2.04, CI: 1.59–2.61); high compared to low CCI-score (IRR = 1.96, CI: 1.16–3.31); HLA-DR mismatch ≥2 vs ≤1 (IRR = 1.54, CI: 1.10–2.18); chronic interstitial nephritis (IRR = 2.53, CI: 1.66–3.85); hypertensive kidney disease (IRR = 1.59, CI: 1.06–2.37); polycystic kidney disease (IRR = 1.66, CI: 1.07–2.57); vasculitis (IRR = 2.61, CI: 1.28–5.30); post-transplant time (compared to 0–6 months: 7–12 months: IRR = 0.39, CI: 0.25–0.59 and 13–24 months: IRR = 0.24, CI: 0.16–0.37 and + 24 months: IRR = 0.18, CI: 0.13–0.24); calendar period (compared to 1990–1995: 2000–2004: IRR = 0.62, CI: 0.41–0.94 and 2005–2009: IRR = 0.42, CI: 0.27–0.64) (Table 3).

**Table 3:** Incidence of first hospitalization for pyelonephritis in renal transplant recipients according to potential risk factors for pyelonephritis

Characteristic	Pyelonephritis Hospitalization, No.	IR^a^ (95 % CI)	Unadjusted IRR (95 % CI)	Adjusted^b^ IRR (95 % CI)	*P* ^*^ Value
Recipient gender					
Male	(122)	13.0 (10.9–15.5)	1 (Reference)	1 (Reference)	
Female	(139)	29.3 (24.8–34.6)	2.25(1.76–2.87)	2.04(1.59–2.61)	<.001
Recipient, CCI-score					
Low (0)	131	15.9 (13.4–18.9)	1 (Reference)	1 (Reference)	
Medium (1–2)	60	17.8 (13.9–23.0)	1.12 (0.83–1.52)	0.98 (0.71–1.36)	.92
High (≥3)	70	27.9 (22.0–35.2)	1.75 (1.31–2.34)	1.96 (1.16–3.31)	.01
Donor type					
Cadaveric donor	190	19.2 (16.6–22.1)	1 (Reference)	1 (Reference)	
Living unrelated donor	22	16.2 (10.7–24.6)	0.85 (0.54–1.32)	1.01 (0.65–1.58)	.95
Living related donor	49	17.2 (13.0–22.8)	0.90 (0.66–1.23)	1.00 (0.71–1.39)	.98
HLA mismatch					
AB mismatch^c^					
<3	122	18.6 (15.5–22.2)	1 (Reference)	1 (Reference)	
≥3	46	16.6 (12.4–22.2)	0.90 (0.64–1.26)	0.88 (0.62–1.25)	.49
DR mismatch^c^					
<2	46	13.6 (10.2–18.1)	1 (Reference)	1 (Reference)	
2	122	20.5 (17.2–24.5)	1.51 (1.07–2.12)	1.54 (1.10–2.18)	.01
Cause of ESRD					
Glomerulonephritis	46	10.3 (7.7–13.7)	1 (Reference)	1 (Reference)	
Diabetes mellitus type 1	53	27.7 (21.2–36.2)	2.69 (1.81–3.40)	1.34 (0.73–2.48)	.35
Diabetes mellitus type 2	4	16.8 (6.33–44.9)	1.64 (0.59–4.55)	1.00 (0.33–2.99)	1.00
Chronic interestitial nephritis	44	31.7 (23.6–42.6)	3.08 (2.04–4.66)	2.53 (1.66–3.85)	<.001
Hypertensive kidney disease	53	17.6 (13.4–23.0)	1.71 (1.15–2.54)	1.59 (1.06–2.37)	.02
Polycystic kidney disease	39	19.2 (14.0–26.3)	1.87 (1.22–2.86)	1.66 (1.07–2.57)	.02
Vasculitis	10	33.2 (17.9–61.7)	3.23 (1.63–6.39)	2.61 (1.28–5.30)	.01
Unkown	12	15.9 (9.0–28.0)	1.55 (0.82–2.92)	1.18 (0.62–2.27)	.62
Posttransplant time					
0–6 months	83	69.9(56.4–86.7)	1 (Reference)	1 (Reference)	
7–12 months	29	26.9(18.7–38.7)	0.38(0.25–0.59)	0.39(0.25–0.59)	<.001
13–24 months	33	16.9 (12.0–23.8)	0.24 (0.16–0.36)	0.24 (0.16–0.37)	<.001
+24 months	116	11.7(9.8–14.0)	0.17 (0.13–0.22)	0.18 (0.13–0.24)	<.001
Recipient age					
<50 years	140	18.2 (15.4–21.5)	1 (Reference)	1 (Reference)	
50–65 years	98	18.7 (15.4–22.8)	1.03 (0.79–1.33)	1.06 (0.80–1.41)	.68
+65 years	23	19.3 (12.8–29.1)	1.06 (0.68–1.65)	1.21 (0.76–1.93)	.43
Calendar period					
1990–1994	35	29.8 (21.4–41.5)	1 (Reference)	1 (Reference)	
1995–1999	67	22.5 (17.7–28.6)	0.76 (0.50–1.14)	0.73 (0.48–1.12)	.16
2000–2004	81	19.1 (15.4–23.7)	0.64 (0.43–0.95)	0.62 (0.41–0.94)	.02
2005–2009	78	13.6 (10.9–17.0)	0.46 (0.31–0.68)	0.42 (0.27–0.64)	<.001

*Abbreviations*: *CI* confidence interval, *ESRD* end-stage renal disease, *HLA* leukocyte antigen, *IR* incidence rate, *IRR* incidence rate ratio, *CCI-score* Charlson co-morbidity index score

^*^ *P*value corresponding to the adjusted IRR

^a^No. of Hospitalizations per 1000 person-years

^b^ Adjusted for all other variables

^c^Missing values not shown

### Mortality and risk of graft loss following hospitalisation for pyelonephritis

In persons with pyelonephritis, 90-day mortality was 1.5 % (CI: 0.58–4.06 %) for RTx recipients and 1.8 % (CI: 0.44–6.08 %) for population controls. The combined risk of graft loss and death in RTx recipients following an episode of pyelonephritis compared to those with no admission due to pyelonephritis was 1.22 fold (CI: 1.01–1.48) (Table 4). In addition, when the first 90 days posttransplantation were censored to eliminate the bias from early graft loss/death following transplantation, an episode of pyelonephritis was associated with a 45 % higher risk of graft loss and death (IRR = 1.45, CI: 1.19–1.77) (Table 4).

**Table 4:** The effect of pyelonephritis on combined endpoint graft function and mortality

Time at risk^a^	No. of Pyelonephritis	IR^b^ (95 % CI)	IRR^e^ (95 % CI)	*p*-value^*^
+ 0 days^c^	0	7.54 (7.10–8.01)	1 (reference)	
	1	9.01 (7.52–10.8)	1.22 (1.01–1.48)	.043
+ 90 days^d^	0	6.41 (6.00–6.85)	1 (reference)	
	1	8.97 (7.49–10.7)	1.45 (1.19–1.77)	≤.001

Abbreviations: No. number, CI confidence interval, IR incidence rate, IRR incidence rate ratio

^*^P value corresponding to the IRR

^a^time of risk = posttransplant time

^b^IR per 100 person-years

^c^Time at risk begins from the day the person is diagnosed with pyelonephritis

^d^The first 90 days post-transplantation are sensored

^e^Adjusted for sex and age

### Validation of discharge diagnosis of pyelonephritis

We reviewed a random sample of 75 medical records with a discharge diagnosis of pyelonephritis. The percentage of episodes recorded in the DNPR fulfilling the criteria for pyelonephritis was 76 % (57/75).

## Discussion

In the present study we found that RTx recipients had a 72-fold higher risk of first-time hospitalisation for pyelonephritis compared to matched population controls. Even when adjusting for potential confounders including calendar period, age, gender and CCI score, the risk of pyelonephritis remained highly elevated during the entire study period in RTx recipients. Although the incidence of hospitalisation for pyelonephritis decreased by more than 50 % during the study period our findings confirm that pyelonephritis is a common cause of hospitalisation after renal transplantation. In addition, we found that the combined risk of graft loss and death was increased by 45 % in RTx recipients following an episode of pyelonephritis compared to those with no admission due to pyelonephritis. These interesting findings highlight the potential for improving the prognosis in renal transplant recipients by further reducing the risk of pyelonephritis.

To our knowledge, this study is among the first to investigate the burden of pyelonephritis among RTx recipients and the impact of pyelonephritis on renal graft survival and mortality in a nationwide population based setting over a 20-year period. The strengths of our study include the use of population-based, nationwide cohorts with inclusion of all adult (from 16-years), first-time RTx recipients, minimal loss to follow-up, long study period, and the availability of comprehensive hospitalisation data. The CCI-score enabled us to adjust for underlying diseases, and the large study size yielded estimates with high statistical precision.

The impact of pyelonephritis on graft and patient survival is debated. While short term mortality was low, we found that hospitalisation for pyelonephritis in RTx recipients was associated with persistent increased risk of graft loss and death. Pellé et al. [16] also found post-transplant pyelonephritis to be an independent risk factor for worse long-term graft survival but found no association with mortality. A study by Giral et al. [17] also suggested that post-transplant pyelonephritis was linked to decreased graft survival. However, this was only the case when pyelonephritis occurred during the first three months post-transplantation. Contrary to Giral et al. a retrospective study by Abbott et al. [18] showed that UTI appearing more than six months after renal transplantation was independently associated with worse long term patient survival and risk of graft loss among RTx recipients. However, this study had some limitations, i.e.,co-morbidities were not taken into account. This may have affected the findings. Thus, it seems difficult to conclude whether UTI was the primary cause of graft loss and death or merely a marker of morbidity.

Chuang et al. [4] also showed that UTI was significantly associated with increased mortality; contrary to Abbott et al. UTI did not increase the risk of renal graft loss. However, the results were not adjusted for co-morbidities and UTI was probably more frequent in the RTx recipients with significant co-morbidities. The above findings are in contrast to Rao KV et al. [19] who showed that late-onset post-transplant UTI is a benign condition.

The above mentioned studies are characterised by somewhat conflicting data. To some degree, this can be explained by “UTI” referring to different conditions in the urinary system, some mainly involving the lower UTIs and some the upper UTIs. Altogether, the majority of studies including ours indicate that severe UTI (e.g., requiring hospitalization) has a negative impact on graft survival and mortality in RTx recipients.

We also found that the incidence of first-time hospitalisation for pyelonephritis among RTx recipients declined throughout the study period. The reason for this finding is not unambiguous but might be explained by early detection of lower UTIs and treatment prior to development of pyelonephritis. In addition, improved per-operative antibiotic prophylaxis, aseptic surgery, invasive procedures and earlier removal of bladder catheters may have contributed. On the contrary, ureteric stents were likely used less frequently at the beginning of the period, but removal of the stents was probably performed earlier by the end of the study period. In addition, there has been a trend towards lower doses of prednisolone in RTx during the study period, which may reduce the risk of UTI. These potential changes could have contributed to the observed changes in IR, but data was not available.

In our study we found no association between diabetes mellitus and increased risk of UTI in RTx recipients; this is in agreement with some previous studies [4, 9, 20] but, whereas others have reported that diabetes mellitus increased the risk of developing UTI in RTx recipients [10, 21, 22]. In our analysis, we only studied the effect of diabetes in patients with diabetes as a cause of ESRD and thus, some patients who had other causes of ESRD may also have had diabetes. However, this proportion of “unregistered” diabetes would likely be low and is unlikely to have a significant effect on the risk estimate. Chuang et al. and Dantes et al. found receiving an organ transplant from deceased donor was independently associated with an increased risk of post-transplant UTI. However, we did not find this association in our present study or in previous studies looking at infection-risk in transplant recipients [23, 24].

Our study also had some limitations. Coding errors may occur in routine hospital discharge data leading to misclassification of pyelonephritis in both RTx recipients and the general population. Differential misclassification may bias the relative risk estimates; however, according to our diagnosis validation, the degree of misclassification was relative low (the positive predictive value of a pyelonephritis discharge diagnosis was 76 % in our study, which is acceptable and in line with other validation studies). While we did not have access to the remaining patients medical records, we consider our validation sample representative for the remaining study population since coding of discharge diagnoses is generally considered uniform throughout Denmark. In addition, doctors may have a lower threshold for hospital admission of RTx recipients with signs and symptoms of pyelonephritis causing us to overestimate the relative risk of pyelonephritis in RTx recipients. In addition, the threshold for hospitalisation may have changed over time in both groups. Another possible limitation of our study was that we lacked information on some potential risk factors for pyelonephritis such as immunosuppressive regimens and the use of ureteral stents. Interestingly, Snyder et al. only found a weak association between steroid use, immune suppressive regimens and first bacterial infections in United States RTx recipients which suggest that the impact of these limitations is limited [25].

## Conclusion

Though the risk of first-time hospitalisation for pyelonephritis declined throughout our study period, pyelonephritis is still a frequent infection after renal transplantation, especially in the first six months post-transplantation and among female transplant recipients. In addition, our findings indicate that pyelonephritis may increase the risk of graft loss and mortality emphasising the importance of preventive measures as well as early detection and treatment of UTI in RTx recipients.

## Abbreviations

CCI, Charlson comorbidity index; CI, confidence interval; CRN, civil registration number; CRS, Danish Civil Registration System; DNPR, The Danish National Registry of Patients; DNR, Danish Nephrology Registry; ESRD, end-stage renal disease; HLA, human leukocyte antigen; IR, incidence rate; IRR, incidence rate-ratio; PYFU, person-years of follow-up; RTx, renal transplant; UTI, urinary tract infection
